# Commentary: The effects of acute stress on core executive functions: A meta-analysis and comparison with cortisol

**DOI:** 10.3389/fpsyg.2017.01711

**Published:** 2017-09-28

**Authors:** Junhua Dang

**Affiliations:** Department of Psychology, Lund University, Lund, Sweden

**Keywords:** stress, executive functions, working memory, inhibition (psychology), task-switching

Recently, Shields et al. conduct a meta-analysis that summarized the effects of acute stress on core executive functions (Shields et al., 2016). It was found that stress impaired working memory and cognitive flexibility. The effect of stress on inhibition was nuanced, such that it impaired cognitive inhibition but enhanced response inhibition. This effortful work advanced our understanding of the relationship between stress and executive functions to a great extant.

While acknowledging its contribution, we suggest that cautious attention has to be paid to the categorizations of tasks measuring inhibition in this paper. Shields et al. (2016) defined response inhibition as “the suppression of a proponent response” and cognitive inhibition as “selectively attending to or ignoring information” (p. 654). Subsequently, as shown in their Table 1, they coded tasks measuring response inhibition and cognitive inhibition. However, after carefully scrutinizing each task, we argue that these categorizations are problematic and thus the conclusion might be misleading.

**Table 1:** Inhibition Tasks included in Shields et al.'s Meta-Analysis.

**Author(s)**	***g***	**95%CI**	**Inhibition task**	**Original coding**	**New coding**
Chajut and Algom, 2003	0.61	[0.27, 0.95]	Stroop	Response inhibition	Response inhibition
Ishizuka et al., 2007	−0.01	[−0.53, 0.50]	Stroop	Response inhibition	Response inhibition
Schwabe et al., 2013	0.53	[−0.12, 1.18]	Stop signal	Response inhibition	Response inhibition
Cackowski et al., 2014	0.21	[−0.18, 0.59]	Go/stop task	Response inhibition	Response inhibition
Finy et al., 2014	0.14	[−0.27. 0.55]	Go/no-go	Response inhibition	Response inhibition
Hendricks, 2013	0.01	[−0.55, 0.57]	Go/no-go	Response inhibition	Response inhibition
Banks et al., 2014	−0.10	[−0.66, 0.46]	SART	Cognitive inhibition	Response inhibition
Vinski and Watter, 2013, Exp. 2	−0.19	[−0.75, 0.38]	SART	Cognitive inhibition	Response inhibition
Vinski and Watter, 2013, Exp. 1	−0.21	[−0.57, 0.15]	SART	Cognitive inhibition	Response inhibition
Alomari et al., 2015	−0.75	[−1.44, −0.06]	SART	Cognitive inhibition	Response inhibition
Cornelisse et al., 2011	−0.11	[−0.79, 0.57]	d2 test	Cognitive inhibition	Cognitive inhibition
Sato et al., 2012	0.18	[−0.70, 1.06]	Flanker	Cognitive inhibition	Cognitive inhibition
Sänger et al., 2014	−0.80	[−1.61, 0.01]	Novel	Cognitive inhibition	Pending
Giles et al., 2015	−0.21	[−0.46, 0.04]	EIT	Cognitive inhibition	Pending
Kuhlmann et al., 2005	0.28	[−0.20, 0.76]	Span forward	Cognitive inhibition	Not inhibition
Quesada et al., 2012	−0.04	[−0.64, 0.56]	Span forward	Cognitive inhibition	Not inhibition
Hoffman and al'Absi, 2004	−0.08	[−0.49, 0.34]	Span forward	Cognitive inhibition	Not inhibition
Schoofs et al., 2009	−0.11	[−0.57, 0.35]	Span forward	Cognitive inhibition	Not inhibition
Taverniers et al., 2010	−0.70	[−1.47, 0.06]	Span forward	Cognitive inhibition	Not inhibition
Mahoney et al., 2007	−0.91	[−1.47, −0.34]	Simple RT and VA	Cognitive inhibition	Not inhibition
McMorris et al., 2006	−0.08	[−0.74, 0.58]	Simple RT	Cognitive inhibition	Not inhibition
Sorg and Whitney, 1992	0.15	[−0.23, 0.53]	Word span	Cognitive inhibition	Not inhibition

*Original coding, Categorizations in Shields et al.'s analysis; New coding, Categorizations in the current analysis; SART, the sustained attention to response task, a typical go/no-go task; EIT, the emotional interference task, which was called as the emotional Stroop task by Shields et al. Simple RT, the simple reaction time task; VA, the visual attention task; Finy et al. (2014), this reference is not included in the reference list because Shields et al. did not report this reference and it could not be found through literature searching*.

First, it was difficult for the reader to follow why the forward span task was coded as a task measuring cognitive inhibition. The span task is in general used to measure working memory. The forward span version requires participants to repeat the sequence of a group of stimuli that were just shown to them. The backward span version requires repeating in reversed order. Correspondingly, the forward span version is assumed to measure temporary storage of working memory whereas the backward span measures the manipulation process of working memory (e.g., Quesada et al., 2012). Shields et al. (2016) did code the backward span task as working memory measurement. However, their coding of the forward span task as cognitive inhibition should be doubted.

In any case, it would be interesting to test whether acute stress influences performance of the forward span task. In Shields et al.'s (2016) paper, there were five studies that used the forward span task. Meta-analysis showed a non-significant result, *g* = −0.06, *Z* = −0.48, *p* = 0.628, 95% CI = [−0.28, 0.17], with low heterogeneity, *Q* (4) = 4.71, *p* = 0.318. Therefore, combined with Shields et al.'s (2016) finding regarding the effect of stress on performance of the *N*-back task and the backward span task, it seems that although stress impairs the manipulation process of working memory, it may not influence temporary storage of working memory.

Second, two studies that used the simple reaction time task (McMorris et al., 2006; Mahoney et al., 2007) were also coded as cognitive inhibition by Shields et al. (2016). In this task, participants were presented with a series of visual stimuli at one of four different locations on the screen and required to indicate the correct spatial location of each stimulus by striking one of four corresponding keys. Mahoney et al. (2007) employed a visual attention task that was also coded as cognitive inhibition. This task required participants to detect a small, faint stimulus that randomly appeared for 1 s at different locations. In addition, Sorg and Whitney (1992) used a word span task that was also coded as cognitive inhibition. This task was very similar to the forward span, in which participants had to recall a set of words in order of presentation. From the task descriptions, it seems apparent that these tasks can hardly be coded as tasks measuring cognitive inhibition.

Third, four studies used the sustained attention to response task (SART). Although with a different name, this task is actually a typical go/no-go task in which participants are required to respond quickly and accurately on non-target trials but withhold response on infrequent target trials. There were also two studies explicitly stated that they used the go/no-go task. Shields et al. (2016) coded these four studies using the SART as cognitive inhibition but coded the two studies using the go/no-go task as response inhibition. If we follow Shields et al.'s (2016) definitions of response inhibition and cognitive inhibition, it might be more suitable to code the go/no-go task as well as the SART as response inhibition. Then we did a meta-analysis by including all the studies using response inhibition tasks, as shown in Table 1, which yielded a non-significant result, *g* = 0.06, *Z* = −0.51, *p* = .611, 95%CI = [−0.17, 0.29], with high heterogeneity, *Q* (9) = 21.11, *p* = 0.012. Even if we restricted the analysis only to studies using the go/no-go task and the SART, the result was still non-significant, *g* = −0.13, *Z* = −1.26, *p* = 0.207, 95% CI = [−0.32, 0.07], with low heterogeneity, *Q* (5) = 5.24, *p* = 0.388. Therefore, it might be safer to conclude that the effect of stress on response inhibition is pending, considering the small number of studies included.

Finally, after excluding abovementioned tasks that were coded as cognitive inhibition by Shields et al. (2016) in an inappropriate way, there were four studies left in the category of cognitive inhibition. Still, whether they can be coded as cognitive inhibition according to Shields et al.'s (2016) definition is debatable. For example, Sänger et al. (2014) employed a novel task that had never been used before, which makes the validity unclear. Giles et al. (2015) used a delayed match-to-sample task with distracters presented during the delay period. The delayed matched-to-sample was coded as a task measuring working memory by Shields et al. (2016). It might be difficult to categorize Giles et al.'s (2015) task as cognitive inhibition just because it included distracters. Although the d2 test of attention (Cornelisse et al., 2011) and the Flanker task (Sato et al., 2012) seem consistent with Shields et al.'s definition of cognitive inhibition, the effect size directions of the studies using these two tasks were opposite, and both effect sizes were negligible. Therefore, the effect of stress on cognitive inhibition should also be pending.

Overall, we suggest unlike working memory and cognitive flexibility, the effect of stress on inhibition, no matter response inhibition or cognitive inhibition, is in need of further investigation.

## Author contributions

The author confirms being the sole contributor of this work and approved it for publication.

### Conflict of interest statement

The author declares that the research was conducted in the absence of any commercial or financial relationships that could be construed as a potential conflict of interest.

## References

[B1] Alomari R. A. Fernandez M. Banks J. B. Acosta J. Tartar J. L. (2015). Acute stress dysregulates the LPP ERP response to emotional pictures and impairs sustained attention: time-sensitive effects. Brain sci. 5, 201–219. 10.3390/brainsci5020201 26010485 PMC4493465

[B2] Banks J. B. Tartar J. L. Welhaf M. S. (2014). Where's the impairment: an examination of factors that impact sustained attention following a stressor. Cogn. Emot. 28, 856–866. 10.1080/02699931.2013.857643 24266660

[B3] Cackowski S. Reitz A. C. Ende G. Kleindienst N. Bohus M. Schmahl C.. (2014). Impact of stress on different components of impulsivity in borderline personality disorder. Psychol. Med. 44, 3329–3340. 10.1017/S0033291714000427 25065373

[B4] Chajut E. Algom D. (2003). Selective attention improves under stress: implications for theories of social cognition. J. Pers. Soc. Psychol. 85, 231–248. 10.1037/0022-3514.85.2.231 12916567

[B5] Cornelisse S. Joëls M. Smeets T. (2011). A randomized trial on mineralocorticoid receptor blockade in men: effects on stress responses, selective attention, and memory. Neuropsychopharmacology 36, 2720–2728. 10.1038/npp.2011.162 21881569 PMC3230495

[B6] Giles G. E. Mahoney C. R. Urry H. L. Bruny,é T. T. Taylor H. A. Kanarek R. B. (2015). Omega-3 fatty acids and stress-induced changes to mood and cognition in healthy individuals. Pharmacol. Biochem. Behav. 132, 10–19. 10.1016/j.pbb.2015.02.018 25732379

[B7] Hendricks M. A. (2013). An Exploration of the Relationship between Emotional and Cognitive Control, Doctoral Dissertation, Saint Louis University.

[B8] Hoffman R. al'Absi M. (2004). The effect of acute stress on subsequent neuropsychological test performance (2003). Arch. Clin. Neuropsychol. 19, 497–506. 10.1016/j.acn.2003.07.005 15163451

[B9] Ishizuka K. Hillier A. Beversdorf D. Q. (2007). Effect of the cold pressor test on memory and cognitive flexibility. Neurocase 13, 154–157. 10.1080/13554790701441403 17786773

[B10] Kuhlmann S. Piel M. Wolf O. T. (2005). Impaired memory retrieval after psychosocial stress in healthy young men. J. Neurosci. 25, 2977–2982. 10.1523/JNEUROSCI.5139-04.2005 15772357 PMC6725125

[B11] Mahoney C. R. Castellani J. Kramer F. M. Young A. Lieberman H. R. (2007). Tyrosine supplementation mitigates working memory decrements during cold exposure. Physiol. Behav. 92, 575–582. 10.1016/j.physbeh.2007.05.003 17585971

[B12] McMorris T. Swain J. Smith M. Corbett J. Delves S. Sale C.. (2006). Heat stress, plasma concentrations of adrenaline, noradrenaline, 5-hydroxytryptamine and cortisol, mood state and cognitive performance. Int. J. Psychophysiol. 61, 204–215. 10.1016/j.ijpsycho.2005.10.002 16309771

[B13] Quesada A. A. Wiemers U. S. Schoofs D. Wolf O. T. (2012). Psychosocial stress exposure impairs memory retrieval in children. Psychoneuroendocrinology 37, 125–136. 10.1016/j.psyneuen.2011.05.013 21696892

[B14] Sänger J. Bechtold L. Schoofs D. Blaszkewicz M. Wascher E. (2014). The influence of acute stress on attention mechanisms and its electrophysiological correlates. Front. Behav. Neurosci. 8:353. 10.3389/fnbeh.2014.00353 25346669 PMC4191471

[B15] Sato H. Takenaka I. Kawahara J. I. (2012). The effects of acute stress and perceptual load on distractor interference. Q. J. Exp. Psychol. 65, 617–623. 10.1080/17470218.2011.648944 22463388

[B16] Schoofs D. Wolf O. T. Smeets T. (2009). Cold pressor stress impairs performance on working memory tasks requiring executive functions in healthy young men. Behav. Neurosci. 123, 1066–1075. 10.1037/a0016980 19824773

[B17] Schwabe L. Höffken O. Tegenthoff M. Wolf O. T. (2013). Stress-induced enhancement of response inhibition depends on mineralocorticoid receptor activation. Psychoneuroendocrinology 38, 2319–2326. 10.1016/j.psyneuen.2013.05.001 23831264

[B18] Shields G. S. Sazma M. A. Yonelinas A. P. (2016). The effects of acute stress on core executive functions: a meta-analysis and comparison with cortisol. Neurosci. Biobehav. Rev. 68, 651–668. 10.1016/j.neubiorev.2016.06.038 27371161 PMC5003767

[B19] Sorg B. A. Whitney P. (1992). The effect of trait anxiety and situational stress on working memory capacity. J. Res. Pers. 26, 235–241. 10.1016/0092-6566(92)90041-2

[B20] Taverniers J. Van Ruysseveldt J. Smeets T. von Grumbkow J. (2010). High-intensity stress elicits robust cortisol increases, and impairs working memory and visuo-spatial declarative memory in Special Forces candidates: a field experiment. Stress 13, 324–334. 10.3109/10253891003642394 20536334

[B21] Vinski M. T. Watter S. (2013). Being a grump only makes things worse: a transactional account of acute stress on mind wandering. Front. Psychol. 4:730. 10.3389/fpsyg.2013.00730 24273520 PMC3824094

